# Genetic polymorphisms and folate status

**DOI:** 10.1111/cga.12232

**Published:** 2017-07-20

**Authors:** Mami Hiraoka, Yasuo Kagawa

**Affiliations:** ^1^ College of Nursing and Nutrition, School of Nutrition Shukutoku University Chiba City Chiba Japan; ^2^ Department of Medical Chemistry Kagawa Nutrition University Sakado City Saitama Japan

**Keywords:** folate, methylenetetrahydrofolate reductase, personalized nutrition, polymorphism

## Abstract

Moderate hyperhomocysteinemia‐induced low folate status is an independent risk factor for cardiovascular disease, dementia, and depression. Folate is an essential cofactor in the one‐carbon metabolism pathway and is necessary in amino acid metabolism, purine and thymidylate synthesis, and DNA methylation. In the folate cycle and homocysteine metabolism, folate, vitamin B12, vitamin B6, and vitamin B2 are important cofactors. Many enzymes are involved in folate transport and uptake, the folate pathway, and homocysteine (Hcy) metabolism, and various polymorphisms have been documented in these enzymes. Serum folate and total Hcy (tHcy) levels are influenced by folate intake and genetic polymorphisms in 5,10‐methylenetertahydrofolate reductase (MTHFR) such as C677T. The prevalence of the MTHFR 677TT genotype varies across ethnic groups and regions, with a frequency of approximately 15% in Japanese populations. Individuals with the TT genotype have significantly higher tHcy levels and lower folate levels in serum than those with the CT and TT genotypes. However, administration of folic acid has been shown to eliminate these differences. Moreover, data have suggested that interventions based on genotype may be effective for motivating individuals to change their lifestyle and improve their nutrition status. Accordingly, in this review, we discuss the effects of MTHFR C677T polymorphisms on serum tHcy and folate levels with folic acid intervention and evaluate approaches for overcoming folic acid deficiency and related symptoms.

## Introduction

The classical symptom of folate deficiency in humans is megaloblastic anemia. Inadequate folate intake constitutes a leading cause of folate deficiency, which involves decreased serum or plasma folate concentrations followed by increased serum or plasma total homocysteine (tHcy) concentrations and reduced red blood cell (RBC) folate levels. Elevated tHcy represents a major risk factor of cardiovascular and cerebrovascular diseases (Homocysteine Studies Collaboration 2002). Moreover, lower serum folate and higher plasma tHcy may also be causes of neural tube defects (NTDs) (Smithells et al. 1976; Daly et al. 1995), cognitive dysfunction (Seshadri et al. 2002), and depression (Bottiglieri 2005).

Folate is an essential cofactor in the folate‐mediated one‐carbon metabolism pathway and is essential in many biochemical processes, such as amino acid metabolism, purine and thymidylate synthesis, and DNA methylation (Brody and Shane 2001). The folate pathway is also closely associated with Hcy metabolism. Hcy itself is located at a branch‐point of metabolic pathways: remethylation and the trans‐sulfuration pathway, in which B‐vitamins, *i.e.* folate, vitamin B12, vitamin B6, and vitamin B2, are required as cofactors. Therefore, inadequate levels of these vitamins can raise plasma tHcy levels.

Several studies have identified associations between genetic polymorphisms related to the folate pathway and Hcy metabolism. The common C677T variant in the gene encoding the folate‐metabolizing enzyme methylenetetrahydrofolate reductase (MTHFR) is the most well‐known genetic factor influencing folate status. MTHFR catalyzes the conversion of 5,10‐methylenetetrahydorfolate to 5‐methyltetrahydrofolate in an irreversible reaction. This enzyme is critical for the regulation of available folate in the remethylation of Hcy. Carriers of the T allele have lower enzyme activity (Frosst et al. 1995), leading to elevated Hcy concentrations (Tsang et al. 2015). The frequency of this polymorphism is known to vary among different ethnic groups and geographical regions (Binia et al. 2014); for example, the allele frequency in the Japanese population is 0.391 (Iida et al. 2009) and the frequency of the homozygous mutant TT genotype is approximately 15% in Japanese individuals (Sadewa et al. 2002; Hiraoka 2004), but about 10% in individuals worldwide (Wilcken et al. 2003). Thus, for prevention of various diseases associated with elevated tHcy, it is important to consider nutrient‐gene interactions.

In this review, we will explore the evidence linking polymorphism‐related folate‐Hcy metabolism with folate status and describe examples of health promotion programs with personalized nutritional intervention based on *MTHFR* C677T polymorphisms.

## Folate and Hcy Metabolism

Dietary folates predominantly exist as polyglutamates, which have to be deconjugated to monoglutamates prior to absorption in order to be transported. The enzyme responsible for this deconjugation in the gut is folyl poly‐γ‐glutamate carboxypeptidase (FGCP), which is anchored to the intestinal apical brush border and is encoded by the glutamate carboxypeptidade II (*GCP1*) gene, also known as folate hydrolase 1 (*FOLH1*) (Chandler et al. 1991). In comparison, synthetic folic acid (pteroylmonoglutamic acid [PteGlu]) has a fully oxidized pteridine ring conjugated to a single glutamate residue.

Folate monoglutamates including PteGlu are absorbed in the duodenum and upper part of the jejunum by the high‐affinity proton‐coupled folate transporter PCFT1 (SLC46A1) (Qiu et al. 2006). However, PteGlu requires an additional step in order to enter folate metabolism, as it must first be reduced to dihydrofolate (DHF) and then to the active form, 5‐methyltetrahydrofolate (5‐methyl‐THF). Once folate has entered the blood stream, 5‐methyl‐THF constitutes the main form as it can enter the cell through folate receptor (FR)‐α. FR‐α is a glycosylphosphatidylinositol‐linked glycoprotein with a high affinity for the monoglutamate 5‐methyl‐THF (Wang et al. 1992) and is expressed in a limited number of epithelial cells, predominantly in the renal proximal tubules, choroid plexus, uterus, and placenta (Kamen & Smith 2004). FR‐β and FR‐γ have a much lower affinity for 5‐methyl‐THF than FR‐α. 5‐Methyl‐THF can also enter the cell by carrier‐mediated transport *via* the ubiquitously expressed reduced folate carrier (RFC), although this has a lower affinity for 5‐methyl‐THF than FR‐α.

**Figure 1:**
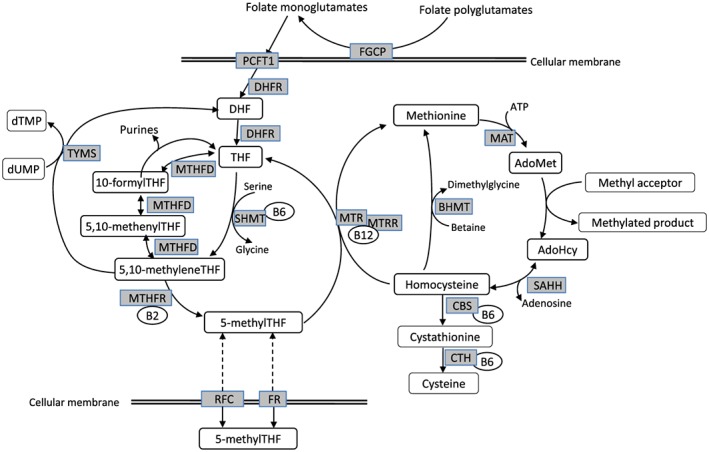
Simplified overview of the folate pathway and homocysteine metabolism. AdoHcy, S‐adenosylhomocysteine; AdoMet, S‐adenosylmethionine; BHMT, betaine‐homocysteine methyltransferase; CBS, cystathionine β‐synthase; CTH, γ‐cystathionase; DHF, dihydrofolate; FGCP, folyl poly‐γ‐glutamate carboxypeptidase; FR, folate receptor; MAT, methionine adenosyltransferase; MTHFD, methylenetetrahydrofolate dehydrogenase; MTHFR, methylenetetrahydrofolate reductase; MTR, methionine synthase; MTRR, methionine synthase reductase; PCFT1, proton‐coupled folate transporter; RFC, reduced folate carrier; SAHH, S‐adenosylhomocysteine hydrolase; SHMT, serine hydroxymethyltransferase; THF, tetrahydrofolate; B2, vitamin B2; B6, vitamin B6; B12, vitamin B12.

After entering the cell, 5‐methyl‐THF functions as a methyl donor for Hcy remethylation (Fig. 1). THF can directly be converted into 5,10‐methylene‐THF by the vitamin B6‐dependent enzyme serine hydroxymethyltransferase (SHMT). Conversion of THF into 5,10‐methylene‐THF *via* 10‐formyl‐THF and 5,10‐methenyl‐THF is catalyzed by the trifunctional enzyme methylene‐THF dehydrogenase (MTHFD), which exhibits formyl‐THF synthase, methenyl‐THF cyclohydrolase, and methenyl‐THF dehydrogenase activities (Hum et al. 1988). 10‐Formyl‐THF can act as a one‐carbon donor for the synthesis of purines. 5,10‐Methylene‐THF can donate a methylene group for the conversion of dUMP into dTMP. This reaction is catalyzed by thymidylate synthase (TYMS) and produces dihydrofolate (DHF), which is reduced back to THF by the action of DHF reductase (DHFR). 5,10‐Methylene‐THF can be further reduced to 5‐methyl‐THF by the riboflavin (vitamin B2)‐dependent enzyme methylene‐THF reductase (MTHFR). This enzyme is critical for the regulation of available 5‐methyl‐THF for Hcy remethylation.

Remethylation of Hcy by methionine synthase (MTR) involves the donation of a methyl group from 5‐methyl‐THF to Hcy, leading to the formation of methionine and THF. MTR requires vitamin B12 (cobalamin) as a cofactor, and the resulting complex, Cbl(I)MTR, binds the methyl group of 5‐methyl‐THF to form methylcbl(III)MTR. The transfer of a methyl group to Hcy results in formation of the Cbl(I)MTR complex. The Cbl(I)MTR complex is also sensitive to oxidation into the active Cbl(II)MTR complex, which can be reactivated to the functional methylcbl(III)MTR by methionine synthase reductase (MTRR) using S‐adenosylmethionine (AdoMet) as a methyl donor. Whereas the MTR enzyme is ubiquitously expressed, another Hcy remethylation system, betaine‐Hcy methyltransferase (BHMT), is mainly expressed in the liver and kidneys.

Methionine adenosyltransferase (MAT) catalyzes the biosynthesis of AdoMet from methionine and ATP. AdoMet is the ultimate donor of methyl groups for methylation reactions; for example, those in DNA, RNA, proteins, and neurotransmitters. Each of these reactions produces S‐adenosylhomocysteine (AdoHcy), an allosteric inhibitor of methylation. AdoHcy is hydrolyzed to adenosine and Hcy by the enzyme AdoHcy hydrolase (SAHH). Because the equilibrium of this reversible reaction favors AdoHcy formation, Hcy and adenosine need to be metabolized to maintain low AdoHcy levels.

In the trans‐sulfuration pathway, Hcy is irreversibly metabolized to cysteine *via* reactions catalyzed by cystathionine β‐synthase (CBS) and γ‐cystathionase, both of which require vitamin B6 (pyridoxal phosphate) as a cofactor.

## Genetic Polymorphisms Related to Folate and Thcy Metabolism and their Effects on Folate Status

In the folate and Hcy metabolic pathways (Fig. 1), MTHFR acts as a key enzyme, as are MTR and CBS, which require B vitamins as cofactors. The genes that encode the proteins/enzymes related to folate uptake and metabolism include many polymorphisms, among which *MTHFR* C677T, *CBS* 844ins68, GCPII H475Y, *MTR* A2756G, and *MTRR* A66G are known to affect folate and vitamin B12 intake (Uauy et al. 2014). Genetic polymorphisms related to the folate pathway have been shown to be associated with functional implications and specific conditions including NTDs (van der Linden et al. 2006), cardiovascular disease (Klerk et al. 2002), and some types of cancers, such as colorectal, breast, and lung cancers (Lee 2009). To date, most studies have shown that the *MTHFR* C677T genotype is related to biomarkers, such as serum folate, tHcy concentration, and folate intake. Other polymorphisms in the folate pathway have been found to be associated with a variety of complex traits and disorders including *MTHFR* A1298C, *MTRR* A66G, *MTR* A2756G, *MTHFD1* G1958A, *FOLH1* T484C, *SLC19A1* A80G, and transcobalamin II (*TCN2*) C776G, the transport protein of vitamin B12 (Weisberg et al. 1998; Gaughan et al. 2001; Laverdiere et al. 2002; Kluijtmans et al. 2003; Dervieux et al. 2004; Martinelli et al. 2006; Moskau et al. 2007; Silva et al. 2011; Pangilinan et al. 2012; Xie et al. 2012). Table 1 summarizes the distribution of serum tHcy, folate, vitamin B6, and vitamin B12 according to polymorphisms related to folate and tHcy metabolism (Hiraoka et al. 2004; Hiraoka et al. 2009).

**Table 1:** Distribution of serum tHcy, folate, vitamin B6, and vitamin B12 concentrations in healthy young Japanese women according to polymorphisms of folate metabolisms

Polymorphism		%	tHcy (μmol/L)	Folate (nmol/L)	Vitamin B6 (μmol/L)	Vitamin B12 (pmol/L)
			(Mean ± SD)			
All subjects (*n* = 250)		100.0	9.1 ± 2.7	18.1 ± 7.5	74.5 ± 65.8	450 ± 154
*MTHFR* C677T rs1801133	CC	32.8	8.8 ± 2.0	20.3 ± 9.3*	66.1 ± 47.5	450 ± 138
	CT	51.6	8.9 ± 2.0	17.3 ± 6.3	69.4 ± 46.7	448 ± 162
	TT	15.6	10.9 ± 4.7*	16.1 ± 5.7	107.3 ± 119.8	474 ± 156
*MTHFR* A1298C rs1801131	AA	68.8	9.4 ± 3.0	17.6 ± 6.1	78.0 ± 70.5	452 ± 157
	AC	29.6	8.6 ± 1.8	19.4 ± 10.1	68.0 ± 55.8	444 ± 151
	CC	1.6	8.2 ± 1.4	17.3 ± 3.8	49.6 ± 11.9	442 ± 24
*MTR* A2756G rs1805087	AA	67.2	9.2 ± 2.2	17.9 ± 7.6	75.4 ± 66.5	459 ± 154
	AG	29.2	9.3 ± 3.6	18.4 ± 6.9	69.0 ± 60.8	421 ± 139
	GG	3.6	7.8 ± 2.1	19.4 ± 11.1	113.5 ± 100.2	513 ± 220
*MTRR* A66G rs1801394	AA	55.6	9.2 ± 3.1	18.2 ± 6.5	72.3 ± 51.1	454 ± 163
	AG	35.9	9.2 ± 2.1	17.9 ± 6.6	84.6 ± 93.0	440 ± 142
	GG	3.6	8.8 ± 2.1	19.2 ± 14.9	52.2 ± 17.3	455 ± 147
*SLC19A1* A80G rs1051266	AA	33.6	9.1 ± 2.6	17.4 ± 5.9	81.5 ± 71.5	438 ± 137
	GA	46.0	9.2 ± 2.9	18.9 ± 8.9	67.0 ± 52.2	473 ± 164
	GG	20.4	9.1 ± 2.2	17.4 ± 6.1	78.0 ± 80.0	415 ± 148
*CBS* 844ins68	DD	99.6	9.2 ± 2.7	18.1 ± 7.5	74.5 ± 66.0	450 ± 154
	ID	0.4	7.7	15.1	69.2	351
GCPII H475Y rs202676	CC	100.0				

Adapted from Hiraoka et al. (2004) and Hiraoka et al. (2009).

* *P* < 0.001: Significantly different from other genotypes.

The common C677T variant in the gene that encodes MTHFR is a C to T transition at position 677, which causes the substitution of alanine with valine. This substitution results in a mildly dysfunctional thermolabile MTHFR enzyme and leads to a 30% decrease in enzyme activity in heterozygotes and a 60% decrease in homozygotes (Frosst et al. 1995). The C677T variant exhibits enhanced loss of the FAD cofactor, creating a thermolabile protein (Yamada et al. 2001) and resulting in decreased 5‐methyl‐THF concentrations and increased tHcy concentrations.

**Figure 2:**
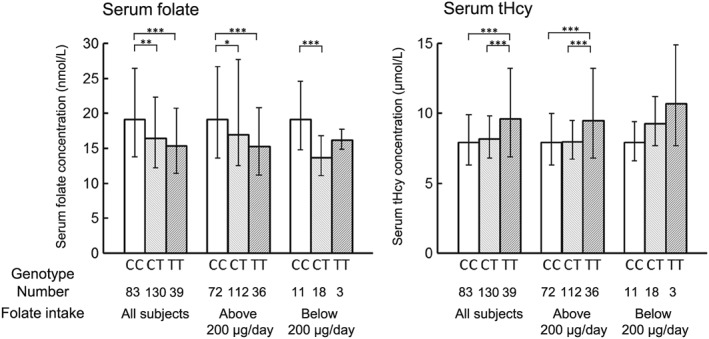
Distribution of serum folate and tHcy concentrations by folate intake according to *MTHFR* C677T genotype in young Japanese women (*n* = 252). The value of 200 μg/day represents the RDA of folate established according to the 6th edition of the RDA for Japanese to be used from April 2000 to March 2005. Values are the geometric mean and bars transformed from the logarithmic‐transformed values of the mean ± SD. Based on data from Hiraoka (2004). Significant difference between genotypes at *P* < 0.05 (^*^), *P* < 0.01 (^**^) and *P* < 0.001 (^***^) for Bonferroni *post hoc* test of analysis of variance.

In a study of Japanese individuals (ages 20–73 years, *n* = 170, TT: 11.8%) with high dietary folate intake (> 255 μg/day) by Nishio et al. (2008), age‐, sex‐, and energy‐adjusted regression analyses revealed that serum folate levels were significantly lower in individuals with the TT genotype than in those with the CC genotype (*P* = 0.01). They suggested that individuals with the TT genotype may need to consume more folate (approximately 1.4 times more) to maintain serum folate levels similar to those found in individuals with the 677CC/CT genotypes. Similar results were obtained in Japanese women (ages 20–22 years, *n* = 252, TT: 15.5%) by Hiraoka (2004). In individuals with folate intake above 200 μg/day, those with the TT genotype exhibited lower serum folate concentrations (*P* < 0.001) and higher serum tHcy concentrations (*P* < 0.001) than those with the CC genotype (Fig. 2). These data suggested that the recommended dietary allowance (RDA) of 240 μg/day for Japanese individuals may not be sufficient for individuals with the TT genotype to maintain serum folate levels and serum tHcy levels similar to those with the 677CC/CT genotype. Moreover, the folate requirement to keep the serum folate levels above 4.4 ng/mL (10 nM), the cutoff point for the appearance of functional pathologies associated with increases tHcy (Selhub et al. 2008), was evaluated based on the equation of plotted folate intake *versus* serum folate: Y (folate intake, μg/day) = 211.314X (logarithmic transformed serum folate, n) + 62.334. The obtained value was 330 μg, after multiplying by 1.2 as the safety margin; this was nearly equal to the mean folate intake. Another study (Taguchi et al. 2012) in young Japanese women (ages 15.4 ± 0.1 years, *n* = 192, TT: 16.1%) also showed that the *MTHFR* C677T polymorphism influenced folate and tHcy status, similar to that in adults, even if the mean concentration of serum folate (4.8 ng/mL, 10.8 nM) in individuals with the TT genotype was lower than that of the overall Japanese population.

Tsang et al. (2015) performed a meta‐analysis of the association between the *MTHFR* C677T polymorphism and blood folate concentrations among women of reproductive age (12–49 years); the percent differences in blood folate concentrations measured by microbiological assays (MAs) between genotypes showed a clear pattern of CC > CT > TT (Table 2). The percent differences in plasma folate were greatest for CC > TT (13%), followed by CC > CT (7%) and CT > TT (6%), and those in RBC folate were in the order of CC > TT (16%), CC > CT (8%), and CT > TT (9%). Plasma folate concentrations measured with protein‐binding assays (PBAs) also showed this pattern, albeit to a greater extent (*e.g.*, CC > TT: 20%). In contrast, RBC folate concentrations measured with PBAs exhibited a clear reverse pattern of CC < CT < TT, as shown by the negative estimated percent differences for genotype comparisons of CC *versus* TT and CT and CT *versus* TT. These data supported previous studies, which also suggested that PBAs may not reflect true whole blood folate concentrations without adjustments for differential folate recovery and *MTHFR* C677T genotype (Fazili et al. 2008). Differential recoveries across assay methods and differential distributions across *MTHFR* C677T genotypes for individual folate species could result in substantial variations in total blood concentrations. *MTHFR* C677T polymorphisms have been shown to alter the mix of circulating folate species in RBCs. Median 5‐methyl‐THF concentrations in individuals with the CC genotype have been reported as 80–100% for RBC folate; in contrast, those in individuals with the TT genotype are only 58–70%. Formyl‐THF species have been observed at up to 59% of the total in those with the TT genotype (Bagley and Selhub 1998). However, no differences in folate species by genotype were found for serum folate (Fazili et al. 2008).

**Table 2:** Blood folate concentrations in women aged 12–49y by *MTHFR* C677T genotypes and assay method from results of the meta‐analysis

Genotype	Serum/Plasma folate, nmol/L		RBC folate, nmol/L	
	MA (*n* = 1590)	PBA (*n* = 10 283)	MA (*n* = 1590)	PBA (*n* = 1146)
CC	15 (10, 25)	15 (14, 17)	694 (546, 939)	468 (304, 687)
CT	14 (10, 23)	14 (12, 15)	639 (504, 863)	484 (315, 708)
TT	13 (9, 22)	12 (11, 13)	579 (455, 783)	531 (345, 783)
Concentration pattern	CC > CT > TT	CC > CT > TT	CC > CT > TT	CC < CT < TT

Values are the estimated median (95% CrI). 95% Equal tailed credible intervals (CrI) defined by the 2.5th and 97.5th percentiles of the posterior distributions for the estimated values. MA, microbiological assay; PBA, protein‐binding assay;

Adapted from Tsang et al. (2015).

Protein‐binding assays rely on folate binding protein (FBP) for detection of the various folate species. Depending on their source, FBPs may have different binding affinities for different folate species. MAs completely recovered folates added to whole blood hemolysates, except for THF, which was recovered at 46.4%. However, PBAs showed poor recovery of 5‐methyl‐THF (51%) and 5‐formyl‐THF (18%) and excellent recovery of THF (152%) (Fazili et al. 2008). Tsang et al. (2015) suggested that researchers should use caution when interpreting RBC folate concentrations assessed by PBAs. Standardizing blood folate assay methods is necessarily in order to avoid compromising interpretation.

The prevalence of the *MTHFR* 677TT genotype varies across ethnic groups and regions. In Europe, Asia, Central America, and South America, the prevalence of the *MTHFR* 677TT genotype ranges from 10% to 32%, whereas different African populations have a prevalence of only 0–3% (Wilcken et al. 2003). Binia et al. (2014) reported that the prevalence rates of the *MTHFR* 677TT genotype were 25% and 57% in Mexican Mestizo and American‐Indian populations, respectively. Yang et al. (2013) showed regional differences in the frequencies of this genotype among Chinese Han populations; the frequencies of the 677TT genotype were significantly higher among northern populations and ranged from 6.4% in Hainan (southern) to 40.8% in Shandong (northern). In Japanese populations, the frequency of the 677TT genotype was found to be approximately 15% (Sadewa et al. 2002; Hiraoka 2004). Thus, these data suggested that the estimated average requirement for folate and the necessity of folic acid and vitamin B supplementation may be increased in some regions around the world.

## Response of Folate Status to Folate Intake and Supplementation

Folate status can be assessed using either serum/plasma folate or RBC folate concentrations, as short‐term and long‐term indicators, respectively. Clinical folate deficiency (associated with megaloblastic anemia) is generally defined as RBC folate concentrations of less than 135 ng/mL (305 nM) and serum concentrations of less than 3.1 ng/mL (7.0 nM) (Green 2008). The cutoff points for the appearance of functional pathologies associated with increased tHcy are greater than 4.4 ng/mL (10 nM) folate in serum and greater than 150 ng/mL (340 nM) folate in RBCs (Selhub et al. 2008). However, RBC folate concentrations should be above 400 ng/mL (906 nM) in women of reproductive age, in order to achieve the greatest reduction of NTDs (Daly et al. 1995; WHO 2015).

**Figure 3:**
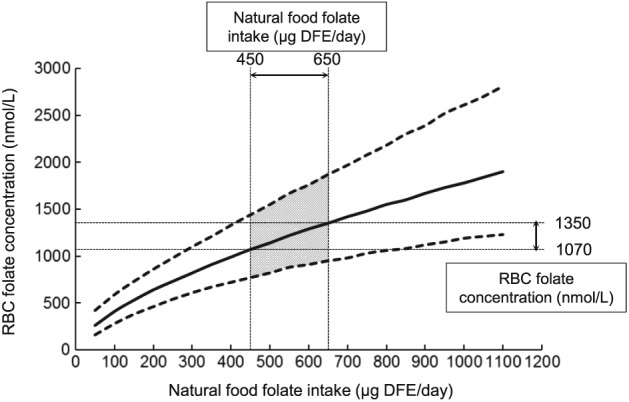
Natural food folate intake (μg/day of DFE) and associated RBC folate concentrations (nmol/L) based on Bayesian modeling of the association between natural food folate intake and RBC folate concentration as analyzed by microbiological assay. Solid line represents the median value under the assumed model. Dotted lines represent the 95% credible interval. The shaded area (natural food intakes between 450 μg DFE/day and 650 g DFE/day) refer to the range of intakes and RBC folate concentrations associated with the lowest population risk for a NTD according to Crider et al. (2014). Based on data from Marchetta et al. (2015). DFE, dietary folate equivalent.

Marchetta et al. (2015) determined the associations between folate intake from natural food alone and blood folate concentrations using a meta‐analysis of studies identified through a systematic literature review (Fig. 3). A 10% increase in natural food folate intake was associated with a 6–7% increase in both serum and RBC folate concentrations. Using modeled results, they estimated that a folate intake of 450 μg dietary folate equivalents (DFE)/day or higher from natural food could achieve the lowest bound of RBC folate concentrations (~1050 nM) associated with a low risk of NTDs.

Both environmental and genetic factors contribute to the development of NTDs. Significantly lower levels of folate are observed in pregnant mothers whose fetuses exhibit NTDs (Kirke et al. 1993) and preconception folic acid supplementation has led to a significant reduction in the risk of NTDs by up to 75% (MRC Vitamin Study Research Group 1991; Czeizel and Dudás 1992; Berry et al. 1999). Genetic factors also contribute to the occurrence of NTDs. The most widely studied genetic risk factor is *MTHFR* C677T polymorphisms. Indeed, a significantly higher frequency of the *MTHFR* 677TT genotype has been observed in cases of NTDs in many populations (Botto and Yang 2000).

The first government‐mandated folic acid fortification program was implemented in the United States of America (USA) beginning in 1998; 79 other countries have also implemented similar programs as of November 2016 (Food Fortification Initiative. 2017). The level of fortification differs among countries; however, in all cases, these programs are designed to reduce the prevalence of pregnancies with NTDs. In the USA and Canada, these folic supplementation programs were thought to be successful because the population folate status was improved (Ray et al. 2002) and reduced rates of NTDs were noted (Honein et al. 2001; Williams et al. 2002), although the results varied depending on ethnicity (Williams et al. 2005). Data from the USA reported from the NHANES trial (Pfeiffer et al. 2012) showed that the mean concentration of serum and RBC folate increased dramatically from the prefortification period (1988–1994) to the postfortification period (1990–2010), from 16.7 ± 0.5 to 41.0 ± 0.3 nM and from 747 ± 10 to 1120 ± 7 nM, respectively, resulting in a 31% reduction in the occurrence of NTDs (Williams et al. 2002).

**Figure 4:**
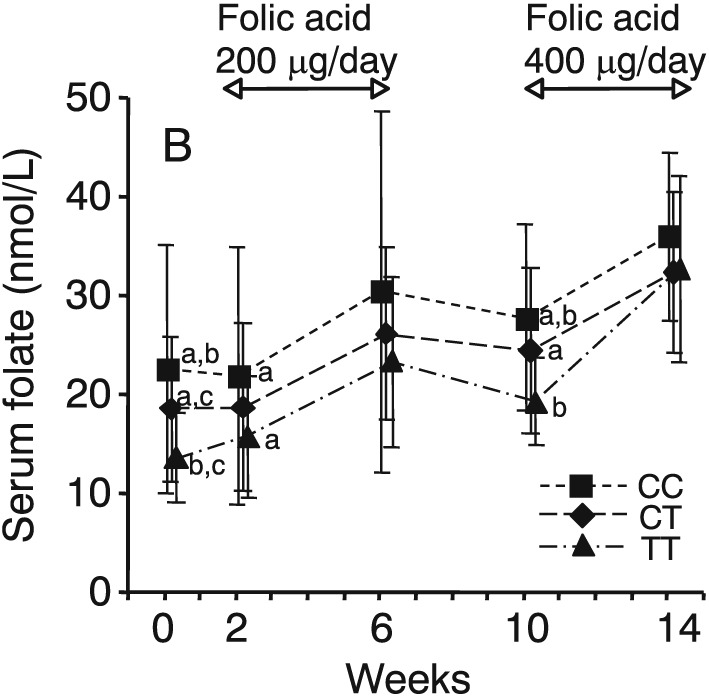
Serum folate concentrations in healthy young Japanese women with differing *MTHFR* C677T genotypes (CC, *n* = 36; CT, *n* = 47; TT, *n* = 17) at baseline and before and after supplementation with 200 and 400 μg/day folic acid. ^a, b, c^ *MTHFR* genotypes with the same superscripts differ significantly; *P* < 0.05. Reproduced from Hiraoka et al. (2004) with permission.

Several studies have reported the effects of the *MTHFR* C677T genotype on the response to folic acid supplementation. A large, population‐based double‐blind folic intervention trial was conducted in northern China by Crider et al. (2011). They examined the response of plasma and RBC folate concentrations and plasma tHcy concentrations to consumption of 100, 400, or 4000 μg folic acid/day or to a single dose of 4000 μg folic acid/week during 3‐ and 6‐month periods after discontinuation of supplementation and assessed responses according to the *MTHFR* C677T genotype. Northern Chinese women (*n* = 932) of childbearing age were enrolled. Plasma and RBC folate and tHcy concentrations were shown to be associated with the *MTHFR* C677T genotype throughout the supplementation trial, regardless of folic acid dose. Within each folic acid dose, the trend of plasma folate concentrations was CC > CT > TT, and that of RBC folate concentrations was CC > TT. Anderson et al. (2013) reported a randomized, double‐blind, controlled, crossover study of different doses of folic acid supplementation and a 30‐week washout period. Volunteers (*n* = 142; ages 18–69 years) were randomized to receive two of three doses (0, 200, or 400 μg/day) of folic acid for a 12‐week period. Serum folate concentrations were responsive to modest increases in folic acid intake, and increases in RBC folate concentrations with 400 μg folic acid supplementation occurred within each *MTHFR* C677T genotype, with individuals with the 677TT genotype having larger responses than those with the CC or CT genotype. Our group (Hiraoka et al. 2004) reported the effects of controlled folate intake among Japanese women (*n* = 100) with various combinations of the four single nucleotide polymorphisms (SNPs) associated with folate metabolism; *i.e.*, *MTHFR* C677T, *MTHFR* A1298C, *MTR* A2756G, and *SLC19A1* A80G. Although there were significant differences in serum folate and tHcy concentrations in individuals with the *MTHFR* C677T genotype at baseline, supplementation with 400 μg/day folic acid for 4 weeks eliminated these differences in individuals with the *MTHRF* C677T genotype (Fig. 4). The effects of the three other SNPs were not as apparent. In a randomized, double‐blind, placebo‐controlled study of healthy male Japanese workers, Miyaki et al. (2005) showed that folic acid supplementation significantly lowered tHcy concentrations for all *MTHFR* C677T genotypes, and the degree to which the tHcy concentration was lowered from baseline was almost the same at 1 and 3 months after supplementation with 1 mg folic acid/day. The effect size of tHcy reduction in the TT genotype was estimated to be 2.4‐fold compared with that in the CC genotype. The inconsistent response of serum and RBC folate or tHcy could be explained by the observation that the baseline folate status appeared to influence changes in these markers (Farrell et al. 2013). These metabolic data regarding folate in individuals with SNPs should be useful for the development of personalized nutrition programs to prevent cardiovascular diseases and dementia.

## Personalized Nutritional Intervention in Folate Status for Individuals with the *MTHFR* C677T Genotype

As described above, folic acid supplementation (400 μg/day) can help overcome the negative health effects of *MTHRF* C677T polymorphisms. Our group implemented the health promotion program designated the “Sakado Folate Project”, which aimed to achieve personalized nutritional intervention based on the *MTHFR* C677T genotype for the prevention of various diseases including cardiovascular diseases and dementia (Kagawa et al. 2017). The project was implemented by a local government in Sakado city and Kagawa Nutrition University, Japan from 2006 to 2015. Participants (*n* = 836, including 179 men and 657 women, age: 62 ± 10 years) were notified of their *MTHFR* C677T genotype and given nutritional guidance. Significant increases in serum folate concentrations and significant decreases in serum tHcy concentrations were observed at 4 months and 1 year after the onset of this intervention. In individuals with the TT genotype, these changes were particularly obvious and intake of green leafy vegetables, which are rich in folate, was increased in individuals with the TT genotype. An increase in the consumption of folic acid‐fortified foods was also observed. These results suggested that intervention of folate status by notification of genotype was effective in motivating individuals to change their lifestyle and improve nutrition status. Similar results were reported in a randomized control trial demonstrating that genotype‐based personalized dietary advice was understood better and was more likely to be followed than general dietary advice (Nielsen and El‐Sohemy 2012).

## Conclusion

*MTHFR* C677T polymorphisms are major factors influencing folate status. Individuals with the TT genotype have lower serum folate concentrations and higher serum tHcy concentrations than those with the CC genotype; hence, folate intake of 400 μg/day above the Japanese current RDA (240 μg/day) is recommended to maintain serum folate and Hcy levels similar to those found in individuals with the 677CC/CT genotypes. Intervention of folate status based on personalized nutrition by notifying individuals of their genotype could be effective in motivating individuals to change their lifestyle and improve their nutrition status, particularly in individuals with the TT genotype. Folic acid‐fortified foods may allow individuals to more easily consume adequate folate to prevent diseases.

## Disclosure

None.
